# A Novel Bacteriophage Infecting Multi-Drug- and Extended-Drug-Resistant *Pseudomonas aeruginosa* Strains

**DOI:** 10.3390/antibiotics13060523

**Published:** 2024-06-03

**Authors:** Guillermo Santamaría-Corral, Israel Pagán, John Jairo Aguilera-Correa, Jaime Esteban, Meritxell García-Quintanilla

**Affiliations:** 1 Clinical Microbiology Department, IIS-Fundación Jiménez Díaz, Universidad Autónoma de Madrid, 28040 Madrid, Spain; guillermo.santamaria@quironsalud.es (G.S.-C.); john.aguilera@fjd.es (J.J.A.-C.); meritxelldejesus@yahoo.es (M.G.-Q.); 2 Centro de Biotecnología y Genómica de Plantas UPM-INIA/CSIC and E.T.S. Ingeniería Agronómica, Alimentaria y de Biosistemas, Universidad Politécnica de Madrid, 28223 Madrid, Spain; jesusisrael.pagan@upm.es; 3 CIBERINFEC-Consorcio Centro de Investigación Biomédica en Red (CIBER) de Enfermedades Infecciosas, 28029 Madrid, Spain

**Keywords:** bacteriophages, phage therapy, *Pseudomonas aeruginosa*, treatment, multidrug-resistance

## Abstract

The prevalence of carbapenem-resistant *P. aeruginosa* has dramatically increased over the last decade, and antibiotics alone are not enough to eradicate infections caused by this opportunistic pathogen. Phage therapy is a fresh treatment that can be administered under compassionate use, particularly against chronic cases. However, it is necessary to thoroughly characterize the virus before therapeutic application. Our work describes the discovery of the novel sequenced bacteriophage, vB_PaeP-F1Pa, containing an integrase, performs a phylogenetical analysis, describes its stability at a physiological pH and temperature, latent period (40 min), and burst size (394 ± 166 particles per bacterial cell), and demonstrates its ability to infect MDR and XDR *P. aeruginosa* strains. Moreover, this novel bacteriophage was able to inhibit the growth of bacteria inside preformed biofilms. The present study offers a road map to analyze essential areas for successful phage therapy against MDR and XDR *P. aeruginosa* infections, and shows that a phage containing an integrase is also able to show good in vitro results, indicating that it is very important to perform a genomic analysis before any clinical use, in order to prevent adverse effects in patients.

## 1. Introduction

*Pseudomonas aeruginosa* is a non-fermenting Gram-negative bacillus and constitutes one of the main opportunistic pathogens causing a wide variety of nosocomial, acute, and chronic infections such as wound and burn infections [1], pneumonia [2,3], septicemia [4], urinary tract [5], and biomaterial-associated infections, especially in immunocompromised individuals. *P. aeruginosa* is one of the most common isolated microorganisms from burn patients, accounting for 57% of positive swabs and tissue cultures [6]. The mortality rates associated with *P. aeruginosa* burn infections with and without bacteremia are 77% and 49%, respectively [7,8]. Likewise, *P. aeruginosa* represents the second most common pathogen in wound infections, associated with 35% of positive wound cultures [9]. The prevalence of this pathogen in diabetic foot ulcers is also notable, reaching 17% in some countries [10,11,12].

A plethora of virulent secreted factors, including proteases, elastases, pyocyanin, exotoxin A, phospholipases, exoenzymes, and cell-associated factors (lipopolysaccharides, flagella, and pili), enable this bacterium to invade host cells and evade host defenses [13]. Furthermore, the failure of antimicrobial treatments is even bigger considering that this bacterium can form biofilms [14], a conglomerate of bacteria surrounded by a self-produced biomatrix [15], intrinsically resistant to a large number of antimicrobials and to phagocytosis [16]. Moreover, *P. aeruginosa* contains a poorly permeable outer membrane and multiple transport systems, providing an innate resistance to many antibiotics such as aminoglycosides, beta-lactams, polymyxins, and quinolones [17]. Apart from its inherent resistance, *P. aeruginosa* can acquire resistance against nearly every type of antibiotic that is available [18]. This resistance has been observed against multiple antimicrobials such as fluoroquinolones, beta-lactams, and aminoglycosides, and there are even versions that are multi-drug resistant (MDR) and extended-drug resistant (XDR) [19]. Changes in efflux pumps, target modifications, beta-lactamases (such as AmpC and carbapenemases), and porin channel modifications are the primary mechanisms that confer resistance in MDR *P. aeruginosa* [20,21]. Resistance determinants can be acquired by the selection of chromosomal gene mutations or by the horizontal absorption of resistance determinants [22]. Mobile genomic islands and integrons that encode carbapenemases or extended-spectrum β-lactamases (ESBLs) commonly co-transferred with aminoglycoside-modifying enzyme determinants, are of particular relevance [23,24]. The World Health Organization (WHO) announced in 2019 that the prevalence of carbapenem-resistant Gram-negative bacteria, including *P. aeruginosa*, has dramatically increased over the last decade [25].

Phage therapy is one of the most promising approaches being explored by researchers to inhibit *P. aeruginosa* strains that are MDR and XDR due to the lack of suitable and effective therapies. Bacteriophages are viruses that enter bacteria, multiply there, and ultimately lyse the bacteria to kill them [26]. Bacteriophage therapy has several benefits over traditional antibiotics, such as the ability to target certain bacterial species, combat antibiotic-resistant species, replicate at the infection site, and remove biofilms [14,26]. Compared to antibiotics, phages have fewer systemic adverse effects because they are species-specific and only affect pathogenic bacteria while leaving harmless commensal bacteria alone [26]. Bacteriophages capable of infecting *P. aeruginosa* have been isolated from hospital sewage, saltwater, ponds, rivers, and wastewater treatment plants [19]. In the mid-1900s, the first phages directed against the *Pseudomonas* genus were reported [27,28].

The current study aims to isolate and characterize the novel bacteriophage vB_PaeP-F1Pa, isolated from sewage water, targeting MDR or XDR clinical *P. aeruginosa* strains. The physical characteristics, environmental stability, whole-genome sequencing, as well as antibiofilm activity, have been demonstrated by this study.

## 2. Materials and Methods

### 2.1. Bacterial Strains and Growth Conditions

*P. aeruginosa* reference strain ATCC15692 (PAO1) was obtained from the American Type Culture Collection (ATCC) (Manassas, VA, USA). Thirty-eight *P. aeruginosa* clinical isolates of patients were donated by the Department of Microbiology, Hospital Universitario Fundación Jiménez Díaz (HUFJD) (Table 1). *P. aeruginosa* clinical isolates were identified using MALDI-TOF (Bruker, VIC, Australia). The *P. aeruginosa* clinical isolates and PAO1 were stored in Difco™ skimmed milk (East Rutherford, NJ, USA) at −80 °C. The clinical isolates were plated from frozen skimmed milk stocks onto tryptic soy agar (TSA) with 5% sheep blood plates (BioMérieux, Marcy l’Etoile, France), and broth cultures were grown in tryptic soy broth (TSB) (BioMérieux, France).

### 2.2. Bacteriophage Isolation

Collection of wastewater samples was done from the sewerage pipelines (receiving fecal matter) of HUFJD. Samples of 50 mL were centrifuged at 900× *g* for 10 min to sediment cellular debris and fecal matter. The supernatant was filtered using a 0.22 µm PES syringe filter (Corning Incorporated, Somerville, MA, USA) to remove bacteria and debris. An amount of 100 µL of filtered solution was combined with 100 µL of the PAO1 overnight culture and 3 mL of molten 0.2% (*w*/*v*) LB agar (Invitrogen, Waltham, MA, USA) (LBA) and plated on 1.5% (*w*/*v*) LBA plates via the double-layer agar method [29]. Following overnight incubation, the obtention of a unique plaque confirmed the presence of phage. An individual plaque was picked using a plastic Pasteur pipette and placed into a 1.5 mL microcentrifuge tube containing 1 mL of sodium magnesium buffer (SM; 100 mM NaCl (Panreac Química, Barcelona, Spain); 10 mM MgSO_4_ (Thermo Fisher Scientific, Waltham, MA, USA); 10 mM CaCl_2_ (Thermo Fisher Scientific, Waltham, MA, USA); 50 mM Tris HCl (Sigma-Aldrich, Merck, Darmstadt, Germany), pH 7.5), and was vortexed vigorously for 5 min and centrifuged at 4000× *g* for 5 min before 4 °C storage of the supernatant.

### 2.3. Bacteriophage Propagation and Titration

A two-step propagation was applied to amplify and purify the isolated phage. For small-scale amplification of phage, 100 µL of PAO1 overnight culture and 100 µL of phage were added to 10 mL of TSB containing 10 mM MgSO_4_ and 10 mM CaCl_2_ and incubated overnight at 37 °C with shaking at 200 rpm. The supernatant containing phage was harvested after centrifugation (900× *g*, 10 min) and bacterial debris was eliminated by filtration (0.22 µm PES syringe filter). Phage titration was performed to calculate the amount of phages.

For large-scale amplification of phage, 500 µL of the PAO1 overnight culture was incubated with 50 mL of TSB for 20 min. After incubation, 100 µL of phage and MgSO_4_ and CaCl_2_ cations were added to obtain a final concentration of 10 mM, and the coculture was incubated overnight at 37 °C with 200 rpm shaking. The phages were harvested as described above on a small scale.

The phage titer was determined via the double-layer agar method [30]. Briefly, phages were serially diluted 1:10 in SM buffer and 100 µL of each dilution of phage and 100 µL of the PAO1 overnight broth culture were added to 3 mL of molten 0.2% (*w*/*v*) LBA containing 10 mM MgSO_4_ and 10 mM CaCl_2_ and overlaid onto a 1.5% (*w*/*v*) LBA plate. The plates were incubated at 37 °C overnight. The phage titer was calculated after counting the calves of the dilutions.

### 2.4. Phage Sequencing and Genomic Annotation

Genome sequencing and bioinformatic analyses for annotation were carried out by AllGenetics & Biology SL (Oleiros, Spain). After sequencing, the complete genome was named vB_PaeP-F1Pa (hereafter in the article the phage will be referred to as F1Pa).

### 2.5. Phylogenetic Tree of the Novel Phage

The closest nucleotide sequences to the new *Pseudomonas* phage were identified using BLASTN. Whole-genome average nucleotide identity (ANI) values between the new *Pseudomonas* phage and the phylogenetically nearest phages were estimated using pANIto (https://github.com/sanger-pathogens/panito; accessed on 5 May 2024). Sequences with a minimum of 70% coverage and sequence identity were selected for analyses of phylogenetic relationships. Maximum likelihood (ML) phylogenies were constructed with 1000 fast bootstrap pseudo-replicates using the TIM2 + F + R2 substitution model, chosen according to the Bayesian Information Criterion (BIC) by ModelFinder [31] as implemented in IQ-TREE v.1.6.12 [32]. The phylogenetic tree was visualized and edited using FigTree v1.4.4 (http://tree.bio.ed.ac.uk/software/figtree/, (accessed on 5 May 2024)).

### 2.6. Temperature and pH Stability

The stability of the F1Pa phage was tested against a wide pH (1–8) and temperature (−80 °C to 60 °C) range using a working stock in TSB with an initial phage titer of around 2 × 10^10^ PFU/mL. Briefly, 10 µL of working stock of each phage was suspended in 1 mL SM buffer previously adjusted with 1 M NaOH or HCl (Sigma-Aldrich, Castle Hill, NSW, Australia) to yield pHs of 1, 4.5, 7.4, and 8. The samples were incubated at room temperature for 1 h. The pH stability testing was studied by plating serial dilutions on LBA plates. For thermal stability, 10 µL of working stock phage was suspended in 1 mL SM buffer and incubated at −80 °C, −20 °C, 4 °C, 21 °C, 37 °C, and 60 °C for 1, 24, and 168 h. The thermal stability testing was studied. For stability in human serum, 10 µL of working stock phage was suspended in 1 mL human serum (Sigma-Aldrich, Merck, Darmstadt, Germany) and incubated at 37 °C for 1, 24, and 168 h. The phage stability for each experiment was determined by measuring the phage titration. This experiment was performed three times using triplicates for each condition.

### 2.7. Adsorption Assays

The adsorption of F1Pa was determined using a methodology previously described [33], which was selected for planktonic testing against the PAO1 reference strain. A volume of 400 µL with 2 McFarland inoculation standards of PAO1 (~10^9^ CFU/mL) was diluted 1:100 in 40 mL of fresh TSB. The MOI of the phages was 0.1. The suspension of bacteria was incubated at 37 °C and 180 rpm. Three ml samples were obtained at 0 min, 1 min, 5 min, 10 min, and then every 10 min for 40 min. For ten minutes, the samples were centrifuged at 715× *g*. The supernatant containing the unabsorbed phages was filtered via a 0.22 µm filter and 1:10 dilutions were plated using the double-layer agar method as previously described for titration. This experiment was performed three times using quadruplicates for each time point.

The adsorption constants were expressed as volume/time (mL/min) and were determined as per the following equation:

$$k=-\ln(P/P_{0})/Nt$$

where *k* is the adsorption rate constant, *P*_0_ and *P* are the starting and ending phage titters, respectively, *N* is the bacterial density, and *t* is the time (min) when adsorption occurred.

### 2.8. One-Step Growth Curve

A one-step growth curve was performed using a methodology previously described [34], with modifications. In 1 mL of TSB, a PAO1 bacterial suspension with a final concentration of 10^9^ CFU/mL and a bacteriophage filtrate with a final concentration of 10^7^ PFU/mL were mixed. The mixture was incubated at 37 °C for 8 min and then centrifuged for 10 min at 1120× *g*. The supernatant was removed, the pellet was resuspended in 100 mL of TSB, and the final suspension was incubated at 37 °C with 180 rpm shaking. Aliquots of 0.5 mL of the resulting suspension were taken every 5 min for 80 min and the bacteriophage titer was assessed using the double-layer agar method. The latency period was defined as the interval between the adsorption of the phage to the host cell and the release of phage progeny. The burst size of the phage was expressed as the ratio of the final count of released phage particles divided by the number of infected bacterial cells during the latency period. This experiment was performed three times using quadruplicates for each time point.

### 2.9. Host Range Analysis

The ability of F1Pa to lyse thirty-eight *P. aeruginosa* clinical strains obtained from HUFJD was tested using spot testing. The host range test was applied. Briefly, 3 μL of the purified phage suspension (10^10^ PFU/mL) and serial dilutions of 1:10 were poured on the surface of the double-layer agar plate previously inoculated with the tested clinical strains. After drying 15 min, the plates were incubated overnight at 37 °C. The host range was determined by visualizing plaques. Three replicates were tested for each bacterial strain.

### 2.10. Inhibition Assays

The infectivity profile of the F1Pa bacteriophage was assessed at MOIs of 0.1, 1, and 10 in liquid. An inoculation of each clinical bacteria (10^9^ CFU/mL) was prepared, and the required volume of phage stock solution (~2 × 10^10^ PFU/mL) was added to the corresponding suspension to achieve MOIs of 0.1, 1, and 10 (n = 10 per concentration) in MicroWell^TM^ flat-bottom 96-well plates (Thermo Fisher Scientific, Waltham, MA, USA). The samples were incubated at 37 °C with a shaking orbital amplitude of 5 mm. Every 5 min for 48 h, the OD_595_ value was measured in a plate reader (Tecan, Männedorf, Switzerland). This experiment was performed in duplicate.

### 2.11. Biofilm Eradication Assays

The F1Pa’s effect on pseudomonal biofilm was determined. Briefly, biofilm formation on the bottom of a MicroWell^TM^ flat-bottom 96-well plate was induced by inoculating 100 µL of Müeller–Hinton broth (MHB) (Sigma-Aldrich, Castle Hill, NSW, Australia) containing 10^6^ CFU/mL of bacteria per well, and the plate was incubated at 37 °C and 5% CO_2_ for at least 18 h [35]. After incubation, the supernatant was discarded, 200 µL per well with different concentrations of F1Pa (n = 16 per concentration) in MHB supplemented with 10 mM of CaCl_2_ and 10 mM of MgSO_4_ were deposited and the plate was incubated at 37 °C and 5% CO_2_ for at least 20 h. After incubation, pseudomonal concentration was determined by measuring the absorbance at 400 nm and bacterial viability was determined by the addition of 200 µL of TSB supplemented with 0.5 mg/mL of 3-(4,5-dimethylthiazol-2-yl)-2,5-diphenyltetrazolium bromide (MTT) (Sigma-Aldrich, Merck, Darmstadt, Germany), and incubating for 1 h at 37 °C, 5% CO_2_, with shaking at 110 rpm. Thereafter, the absorbance at 570 nm was measured. This experiment was performed in triplicate. The formula used to calculate the percentage of biofilm inhibition was as follows:

$$Biofilm\;inhibition=\frac{Abs\;Treatment\;Group-Abs\;Control\;Group}{Abs\;Control\;Group}$$

### 2.12. Inhibition of Biofilm Formation

The quantification of the inhibition of biofilm by bacteriophage was performed in microtiter plates as previously described [36], with modifications. Briefly, biofilm formation on the bottom of a MicroWell^TM^ 96-well flat-bottom plate was induced by inoculating 100 µL of TSB supplemented with 1% (*w*/*v*) glucose, 10 mM of CaCl_2_, and 10 mM of MgSO_4_ containing 10^6^ CFU/mL of bacteria and 100 µL with different concentrations of F1Pa (n = 24 per concentration) in TSB supplemented with 1% glucose (Sigma-Aldrich, Merck, Darmstadt, Germany), 10 mM of CaCl_2_, and 10 mM of MgSO_4_ per well. The positive control wells contained only broth: 200 μL of TSB supplemented with 1% glucose, 10 mM of CaCl_2_, and 10 mM of MgSO_4_, containing 10^6^ CFU/mL of bacteria per well. The plate was incubated at 37 °C and 5% CO_2_ for at least 18 h. After incubation, the medium was removed and the wells were washed once with sterile saline (0.9% NaCl) (Fresenius, Bad Homburg, Germany). The medium was then removed, and fixation was done with 150 μL of methanol for 20 min. After fixation, methanol was removed, and microtiter plates were dried at room temperature for 10 min. A volume of 200 μL of safranin (BioMérieux, France) was added to each well and left for 15 min at room temperature. The dye was then removed, and this was followed by 2 washings with 250 μL sterile distilled water. The dye bound to the cells was resolubilized with 200 μL of 95% ethanol per well and, thereafter, the microtiter plate was covered with the lid and was dried at room temperature for at least 5 min without shaking. Biofilm formation was determined by measuring the absorbance at 492 nm. This experiment was performed in triplicate. The formula to calculate the percentage of biofilm formation inhibition was as follows:

$$Biofilm\;formation\;inhibition=\frac{Abs\;Treatment\;Group-Abs\;Control\;Group}{Abs\;Control\;Group}$$

### 2.13. Statistical Analysis

All statistical analysis was performed using R (R Core Team, 2017) with R commander, except for linear regressions that were carried out using GraphPad Prism v.8 (GraphPad Prism, version 8.0.1 (86); Windows Version by Software MacKiev © 2020-2018 GraphPad Software, LLC.; San Diego, CA, USA) and STATA statistical software, release 11 (StataCorp, 2009, StataCorp LP., College Station, TX, USA). Data distribution was evaluated using Shapiro–Wilk or Kolmogorov–Smirnov statistics. Descriptive statistics are cited as the median and interquartile range (non-normal distribution) for each variable that was calculated. A non-parametric Mann–Whitney test considering equality of variances was used to compare two groups, and a non-parametric Kruskal–Wallis test was used to compare more than two groups. To determine the effect of temperature on phage viability, bacteriophage adsorption, and burst size, data were analyzed using linear regression. Bacteriophage inhibition of bacterial biofilm was analyzed by Dunn’s pairwise test with a Benjamini–Hochberg procedure. The possible relation between the bacterial biofilm or planktonic concentration and the concentration of phage was determined by using the Spearman rank correlation coefficient. The significance level was established at α = 0.05.

## 3. Results

### 3.1. Comparative Genomics

The genome size of the vB_PaeP-F1Pa bacteriophage was 62,345 nucleotides. The complete phage genome sequence was deposited in GenBank under accession number PP735386. PhageTerm did not locate any terminal sequence, and Li’s method indicated ends with redundant sequences, which is generally typical of circularizing phages.

The sequence included a region coding for an integrase, which is typical of temperate bacteriophages. The vB_PaeP-F1Pa bacteriophage genome encoded for an integrase (nucleotides 4113–5219, 368 aa), with 100% amino acid sequence homology with a functional phage integrase obtained from Pseudomonas aeruginosa (acc. N. WP_019485068). It is also highly similar to other phage integrases also obtained from the same bacteria that differ in a single residue (acc. N. WP_033937549, 99% amino acid sequence homology). Interestingly, this mutated residue falls outside of the fimB and DNA_BRE_C conserved domains, and, thus, likely has little effect on protein function (Figure S1). These results strongly suggest that the vB_PaeP-F1Pa integrase is functional.

**Figure 1:**
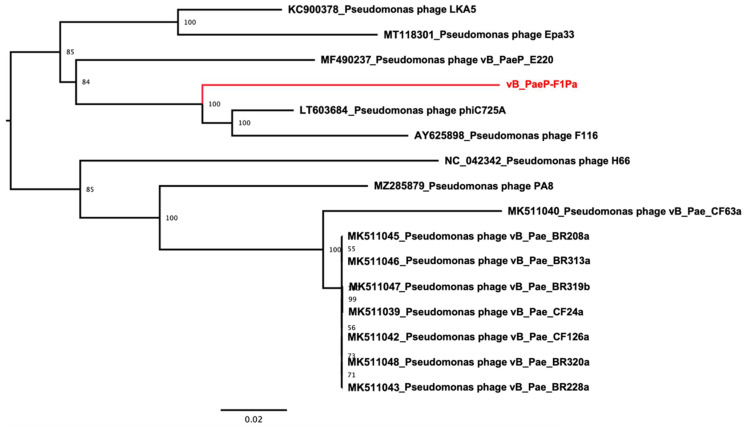
Maximum likelihood phylogenetic tree of the whole-genome sequence of members of the genus *Hollowayvirus*. Numbers indicate bootstrap values in percentages (1000 pseudo-replicates). The *Pseudomonas* phage isolated and characterized here is indicated in red. The tree is midpoint rooted.

Following the criteria described in the Materials and Methods section, fifteen nucleotide sequences were identified as the nearest to the new *Pseudomonas* phage (Figure 1). All these sequences belonged to the genus *Hollowayvirus* and included the type species for this genus (*Pseudomonas* virus H66, NC_042342). All identified sequences showed pairwise nucleotide identities ranging from 87% to 93%, which is within the interval of nucleotide identities shared by the other members of the genus *Hollowayvirus*. Hence, our data support that the new *Pseudomonas* phage belongs to this genus and, due to its nucleotide identity being less than 95%, it constitutes a new bacteriophage [37].

### 3.2. Temperature and pH Stability

**Figure 2:**
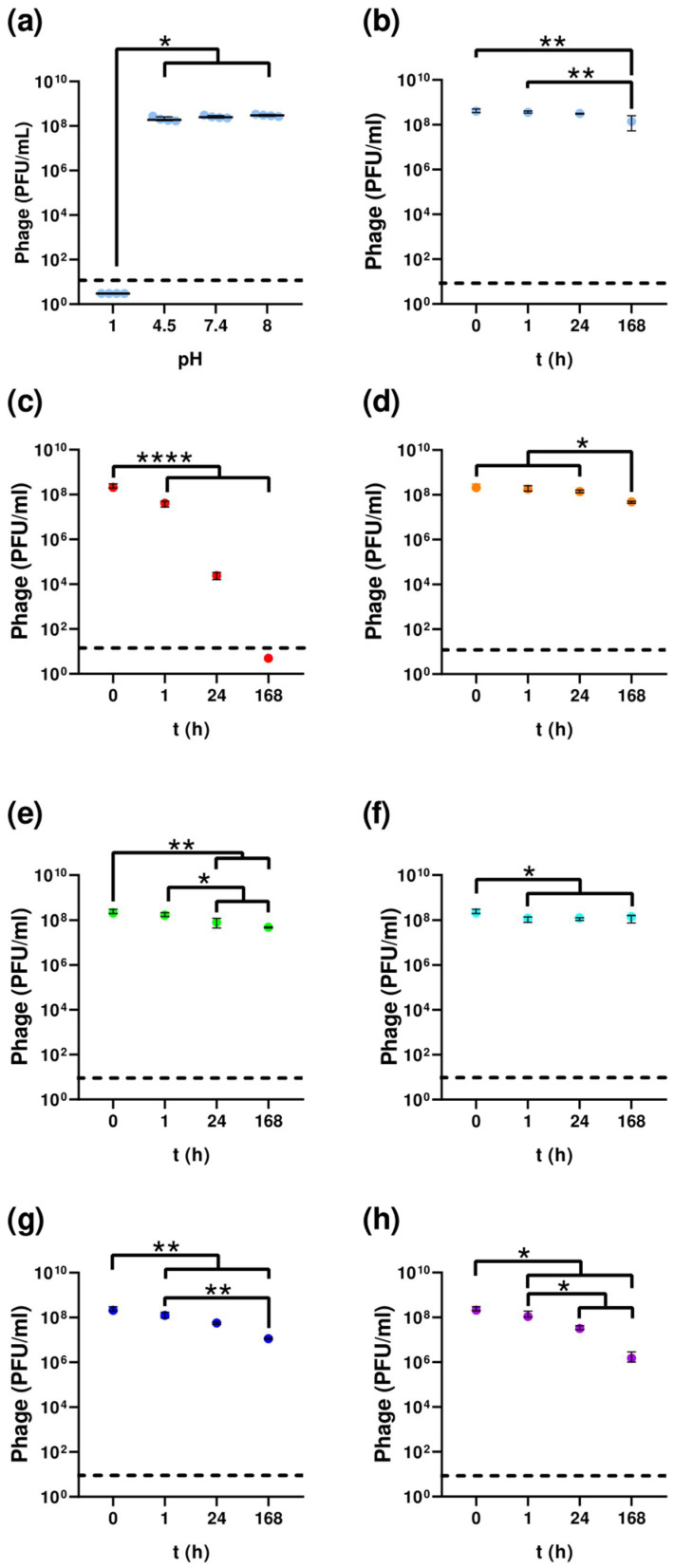
F1Pa pH (**a**), in human serum (**b**), and thermal stability at different temperatures: 60 °C (**c**), 37 °C (**d**), 21 °C (**e**), 4 °C (**f**), −20 °C (**g**), and −80 °C (**h**). The bar represents the median and the interquartile range. Discontinued lines denote limit detection of quantification. *: *p*-value < 0.05, **: *p*-value < 0.01, ****: *p*-value < 0.0001.

F1Pa was tested against a wide pH range (pHs 1, 4.5, 7.4, and 8), for a one-hour incubation period to establish its stability under acidic and alkaline conditions (Figure 2a). After 1 h of incubation at a pH of 1, no plaques were detected, suggesting no active phage (*p*-value < 0.05). At the other pH values tested, F1Pa was stable.

In human serum at 37 °C (Figure 2b), the F1Pa titer was reduced by only 0.36% (R^2^ = 0.6329, *p*-value > 0.001) compared to the initial concentration, and, therefore, is considered stable in human serum.

F1Pa was stable at 4 °C and did not lose viability after 1 h, 24 h, and 168 h of incubation (Figure 2c). At 60 °C, 37 °C, 21 °C, −20 °C, and −80 °C, the phage titer was reduced over time only by 6% (R^2^ = 0.9099, *p*-value > 0.0001), 0.45% (R^2^ = 0.8953, *p*-value > 0.0001), 0.40% (R^2^ = 0.5931, *p*-value > 0.001), 0.8% (R^2^ = 0.8659, *p*-value > 0.0001), and 1.4% (R^2^ = 0.9071, *p*-value > 0.0001) with respect to the initial concentration, respectively.

### 3.3. Adsorption Assays

**Figure 3:**
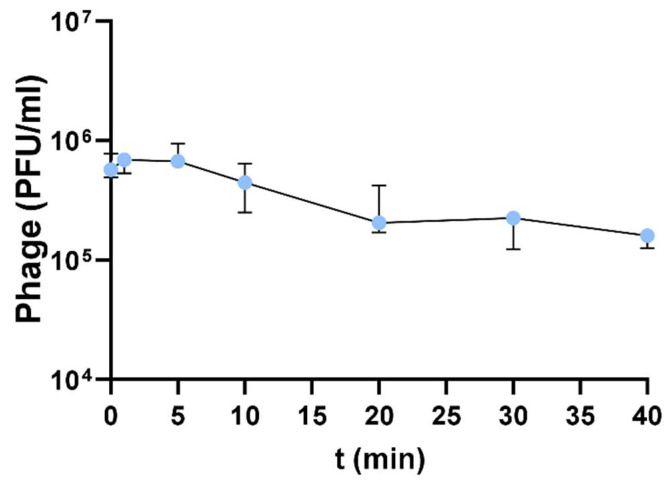
Determining the adsorption of F1Pa to the host bacterial surface. The bars represent the median and the interquartile range.

The bacteriophage planktonic binding properties were determined through an adsorption assay (Figure 3). F1Pa showed strong adsorption, with isolates being adsorbed at a rate of 3% of phage titer (PFU/mL) per minute (R^2^: 0.7446, *p*-value < 0.001). The adsorption experiment lasted 40 min, and the adsorption rate constant was calculated as in previous studies [38]. The adsorption rate constant of F1Pa was about 4.20 × 10^−9^ mL/min at 20 min. Statistically significant differences were observed in the amount of bacteriophages absorbed at the different time points (Table S1).

### 3.4. One-Step Growth Curve

**Figure 4:**
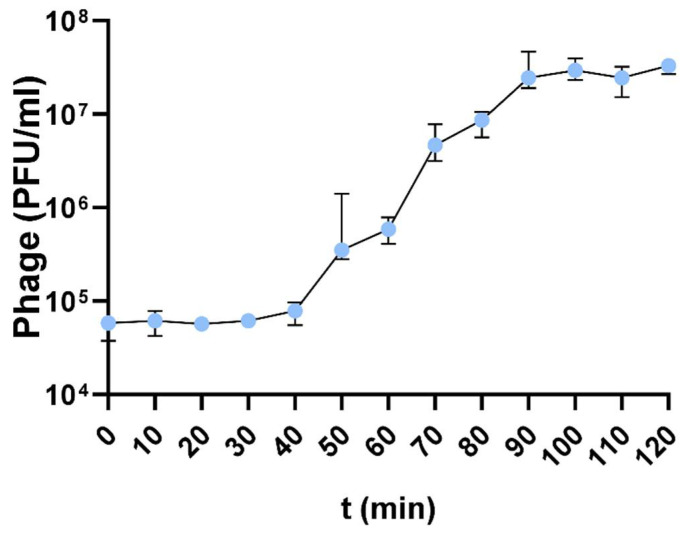
One-step growth curve of F1Pa phage. The bars represent the median and the interquartile range.

Based on the one-step growth curve experiment, the latent period and burst size for the F1Pa phage were calculated (Figure 4). The latent period was 40 min for F1Pa. The burst size was 394 ± 166 particles per bacterial cell. Until 40 min into the experiment, bacteriophage particles were released (R^2^ = 0.0223, *p*-value = 0.4151). However, between 40 and 90 min, the release of bacteriophage particles followed a lineal tendency, increasing by 30% PFU/mL·min (R^2^= 0.9292, *p*-value < 0.0001). Statistically significant differences were observed in the amount of phage particles released at the different time points (Table S2).

### 3.5. Host Range Analysis

The host range against thirty-eight *P. aeruginosa* clinical isolates for phage F1Pa was 23/38 susceptible (+) and 15/38 non-susceptible (−) strains. Because the strains from this collection were not sequenced, we could not conclude that F1Pa exhibits a broad host range, but we can say that this phage would have been able to infect 23 out of 38 of the clinical isolates from the hospital FJD from different patients with specific antimicrobial susceptibility and from diverse samples (Table 1). Within those 23 sensitive strains, there were nine MDR *P. aeruginosa* strains (which include PA24, PA35, and PA36) and five XDR (which include PA35) strains. More than 70% of the clinical isolates were from respiratory and wound samples, and the bacteriophage had lytic activity against 43% and 92% of them, respectively.

### 3.6. Inhibition Assays

**Figure 5:**
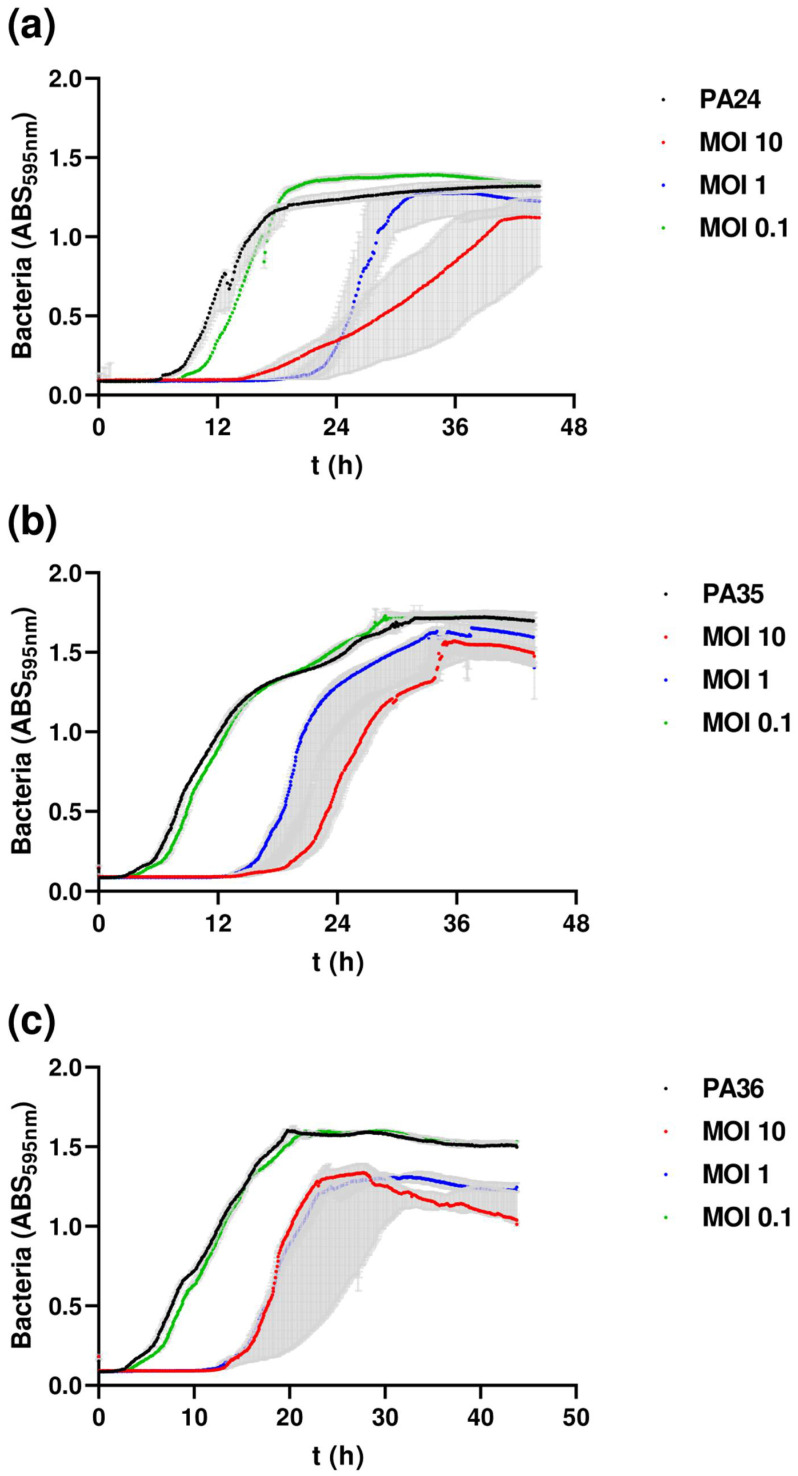
Bacteriophage inhibition assays against three MDR clinical isolates PA24 (**a**), PA35 (**b**), and PA36 (**c**). The grey bars represent the interquartile range (Q1–Q3).

The F1Pa planktonic infective properties were determined through inhibition assays (Figure 5). The phage inhibition of clinical isolates was assessed for 48 h when infected at multiplicities of infection (MOIs) of 10, 1, and 0.1 for PA24 (Figure 5a), PA35 (Figure 5b), and PA36 (Figure 5c). F1Pa inhibited bacterial growth at MOIs 10 and 1 at between 12 and 24 h in the three *P. aeruginosa* clinical strains. Statistically significant differences were observed in bacterial growth of the three clinical isolates of *P. aeruginosa* according to the MOI of bacteriophage F1Pa at 6, 12, 24, and 36 h (Tables S3–S14).

### 3.7. Effect of Phage on Preformed Biofilm

Initially, biofilms of the *P. aeruginosa* clinical strains tested in the study were characterized. The median optical density (OD) value, 4.08 (1.58 to 7.81), showed a broad distribution between the different strains (Table 2). According to this classification, most of the strains studied were biofilm producers (94%). From 38 bacterial isolates tested from biofilm formation, 20 were categorized as strong producers, 5 were categorized as moderate producers, 11 were categorized as weak producers, and 2 were categorized as non-biofilm producers. Regarding the biofilm experiments, we chose *P. aeruginosa* clinical strains PA24, PA35, and PA36 because they were susceptible to phage F1Pa, strong biofilm producers, and MDR, including to beta-lactam antibiotics.

**Figure 6:**
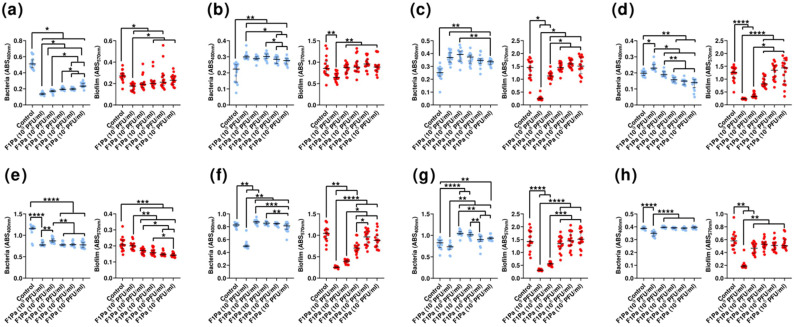
F1Pa’s effect on PAO1, PA24, PA35, and PA36 strains’ biofilm growth in both planktonic state (blue) and biofilm form (red) at 6 h (**a**–**d**) and 24 h (**e**–**h**), respectively. The bars represent the median and the interquartile range. *: *p*-value < 0.05, **: *p*-value < 0.01, ***: *p*-value < 0.001, ****: *p*-value < 0.0001 for Dunn’s pairwise test.

The bacteriophage’s effect at 6 and 24 h of treatment on preformed 24 h *P. aeruginosa* biofilm was determined by quantifying the bacteria present in the planktonic and biofilm states. The concentration of PAO1 in the planktonic bacteria from the biofilm decreased by 63% in the presence of any concentration of bacteriophage (*p*-value < 0.05) at 6 h (Figure 6a). The quantity of planktonic bacteria derived from the biofilm and the quantity of bacteriophages showed a strong negative correlation (ρ = −0.9168, *p*-value < 0.0001) at 6 h. Concentrations of 10^9^, 10^8^, 10^7^, and 10^6^ PFU/mL were able to reduce by 35%, 29%, 26%, and 22% the amount of PAO1 biofilm growth, respectively, (*p*-value < 0.05) at 6 h (Figure 6a). The amount of biofilm and the concentration of phage showed a moderate negative correlation (ρ = −0.5354, *p*-value < 0.0001) at 6 h. Concentrations of 10^9^, 10^7^, 10^6^, and 10^5^ PFU/mL of F1Pa were able to reduce the concentration of PAO1 in the planktonic bacteria from the biofilm by 32% (*p*-value < 0.0001) at 24 h (Figure 6e). The concentration of planktonic bacteria from the biofilm and the concentration of bacteriophage showed a weak positive correlation (ρ = 0.2342, *p*-value = 0.0216) at 24 h. The concentrations of 10^8^, 10^7^, 10^6^, and 10^5^ PFU/mL of F1Pa reduced the amount of PAO1 biofilm by 27% (*p*-value < 0.001) at 24 h (Figure 6e). There was no correlation between the amount of biofilm and the bacteriophage concentration (*p*-value = 0.107) at 24 h.

The concentration of PA24 clinical isolate in the planktonic bacteria from the biofilm increased by 30% in the presence of any concentration of bacteriophage (*p*-value < 0.01) at 6 h (Figure 6b). The concentrations of PA24 and F1Pa showed a strong positive correlation (ρ = 0.6545, *p*-value < 0.0001) at 6 h. Only the concentration of 10^9^ PFU/mL was able to reduce the amount of PA24 biofilm by 27% (*p*-value < 0.01) at 6 h (Figure 6b). The amount of biofilm and phage showed a weak negative correlation (ρ = −0.3491, *p*-value < 0.0001) at 6 h. Similarly, 10^9^ PFU/mL F1Pa was able to reduce the concentration of PA24 in the planktonic bacteria from the biofilm by 37% (*p*-value = 0.0003) at 24 h (Figure 6f). There was no correlation between planktonic bacteria from the biofilm and bacteriophage concentration (*p*-value = 0.2532) at 24 h. Concentrations 10^9^, 10^8^, and 10^7^ PFU/mL F1Pa reduced by 76%, 62%, and 33% the amount of PA24 biofilm, respectively, (*p*-value < 0.01) at 24 h (Figure 6f). The bacteriophage concentration and the amount of biofilm showed a very strong negative correlation (ρ = −0.8439, *p*-value < 0.0001) at 24 h.

The concentration of PA35 clinical isolate in the planktonic bacteria from the biofilm increased by 29% in the presence of any concentration of bacteriophage (*p*-value < 0.01) at 6 h (Figure 6c). The concentration of planktonic bacteria from the biofilm and bacteriophage showed a strong positive correlation (ρ = 0.6164, *p*-value < 0.0001) at 6 h. Concentrations of 10^9^ and 10^8^ PFU/mL F1Pa were able to reduce the amount of PA35 biofilm by 83% and 23%, respectively (*p*-value < 0.05) at 6 h (Figure 6c). The amount of biofilm and F1Pa concentration showed a moderate negative correlation (ρ = −0.5980, *p*-value < 0.0001) at 6 h. Concentrations of 10^8^, 10^7^, and 10^5^ PFU/mL of F1Pa were increased by 29%, 25%, and 16% the concentration of PA35 in the planktonic bacteria from the biofilm, respectively, (*p*-value < 0.01) at 24 h (Figure 6g). There was no correlation between the concentration of planktonic bacteria from the biofilm and F1Pa concentration (*p*-value = 0.3937) at 24 h. Concentrations of 10^9^ and 10^8^ PFU/mL F1Pa reduced the amount of PA35 biofilm by 78% and 61%, respectively, (*p*-value < 0.0001) at 24 h (Figure 6g). The amount of biofilm and F1Pa concentration showed a strong negative correlation (ρ = −0.7515, *p*-value < 0.0001) at 24 h.

The concentration of PA36 in the planktonic bacteria from the biofilm decreased by 21%, 25%, and 29% in the presence of 10^7^, 10^6^, and 10^5^ PFU/mL F1Pa concentrations, respectively (*p*-value < 0.01), at 6 h (Figure 6d). The concentration of planktonic bacteria from the biofilm and the concentration of bacteriophage showed a moderate positive correlation (ρ = 0.4463, *p*-value < 0.0001) at 6 h. Concentrations of 10^9^ and 10^8^ PFU/mL F1Pa were able to reduce the amount of PA36 biofilm by 84% and 74%, respectively (*p*-value < 0.0001), at 6 h (Figure 6d). The amount of biofilm and the concentration of F1Pa showed a strong negative correlation (ρ = −0.7908, *p*-value < 0.0001) at 6 h. Only the 10^9^ PFU/mL F1Pa concentration was able to significantly reduce the concentration of PA36 in the planktonic bacteria from the biofilm, by 10% (*p*-value < 0.0001) at 24 h (Figure 6h). The concentration of planktonic bacteria from the biofilm and the concentration of F1Pa showed a weak negative correlation (ρ = −0.3091, *p*-value = 0.0022) at 24 h. Concentrations 10^9^ and 10^8^ PFU/mL F1Pa reduced the amount of the PA36 biofilm by 68% and 20%, respectively (*p*-value < 0.01), at 24 h (Figure 6h). The amount of biofilm and the concentration of F1Pa showed a strong negative correlation (ρ = −0.6018, *p*-value < 0.0001) at 24 h.

### 3.8. Inhibition of Biofilm Formation

**Figure 7:**
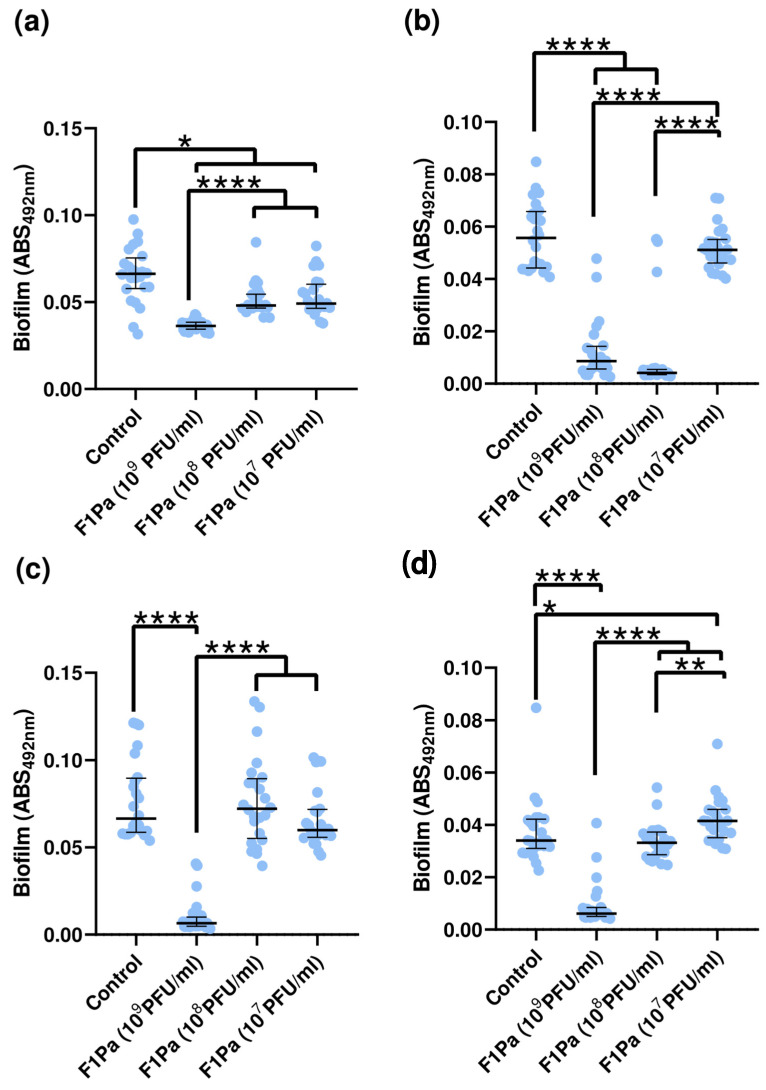
F1Pa’s effect on biofilm formation of PAO1 (**a**), PA24 (**b**), PA35 (**c**), and PA36 (**d**) strains at 24 h. The bars represent the median and the interquartile range. *: *p*-value < 0.05, **: *p*-value < 0.01, ****: *p*-value < 0.0001 for Dunn’s pairwise test procedure.

The ability of bacteriophage F1Pa to prevent biofilm formation of three clinical and one reference strains of *P. aeruginosa* was tested. The formation of biofilm of PAO1 was 30% lower in the presence of any concentration of bacteriophage (*p*-value < 0.05 for the three concentrations evaluated) compared to the positive control (Figure 7a). The formation of biofilm of PAO1 and concentration of F1Pa showed a strong negative correlation (ρ = −0.7103, *p*-value < 0.0001). Concentrations of 10^9^ and 10^8^ PFU/mL F1Pa were able to reduce the formation of biofilm of clinical PA24 by 83% and 92% (*p*-value < 0.0001 for both concentrations), respectively, compared to the positive control (Figure 7b). The formation of biofilm of PA24 and concentration of F1Pa showed a strong negative correlation (ρ = −0.7022, *p*-value < 0.0001). The only concentration of F1Pa that could diminish the formation of PA35 biofilm was 10^9^ PFU/mL, by 90% (*p*-value < 0.0001) compared to the positive control (Figure 7c). The formation of biofilm of PA35 and concentration of F1Pa showed a moderate negative correlation (ρ = −0.5867, *p*-value < 0.0001). The concentration of 10^9^ PFU/mL F1Pa was able to reduce the formation of biofilm of PA36 by 82% (*p*-value < 0.0001) compared to the positive control (Figure 7d). The formation of biofilm of PA36 and concentration of F1Pa showed a strong negative correlation (ρ = −0.6215, *p*-value < 0.0001).

## 4. Discussion

In the present study, the specific bacteriophage vB_PaeP-F1Pa against *P. aeruginosa* was isolated from wastewater. This medium is often contaminated by a wide range of microorganisms from the hospital, as well as fecal wastes and bacteriophages [19].

The term “host range” refers to the variety of organisms that a parasite can infect; host, parasite, or environmental factors might place restrictions on the host range [39]. A total of 38 *P. aeruginosa* strains isolated from different locations of infected patients were used to determine the host range of F1Pa. F1Pa exhibited activity to lysate 60.5% (23/38) of the clinical *P. aeruginosa* strains evaluated here. The data about the lytic activity of F1Pa are similar to data previously described for other *Podoviridae* bacteriophages such as Lx18 [40], vB_PaEP_PAO1EW [41], and δ phages [42], which were able to lyse about 70% of *P. aeruginosa* strains within the *P. aeruginosa* clinical strains tested in the host range assay; 56% (n = 16) are MDR and 18% (n = 7) are XDR. F1Pa was active against 56% (9/16) of MDR *P. aeruginosa* clinical strains tested, which is consistent with the previous study that described RLP bacteriophage lytic activity against 50% (19/38) of MDR *P. aeruginosa* clinical strains [43]. Despite its lytic activity, this bacteriophage cannot be classified as a lytic phage because an integrase has been found in its sequence. It is important to know that even when an integrase is coded in the genome, a bacteriophage can have promising results in vitro, meaning that a previous in silico analysis of the sequence is essential before any compassionate use. This kind of phage may be involved in transduction, which is not desirable at all [44,45,46,47]. The use of this type of phage is undesirable for treating infected patients mainly for three reasons. i. Integrated phages may spread pieces of bacterial DNA, including antibiotic-resistant genes and virulence genes, to other bacteria; ii. the integration of temperate phages may induce bacteria to become resistant to previous sensitive antibiotics as a consequence of the site of insertion; iii. the lysogenic state avoids the invasion by other phage viruses [48]; all these events would cause adverse effects on the patients, making it even more difficult to treat the infection. Our results should alert other authors to these aspects, which must be taken into account when selecting new phages to inactivate pathogenic bacteria. Lytic phages must be chosen for clinical use. Moreover, a lytic derivative achieved through genetic engineering is mandatory when a temperate phage is needed if only a scarce number of phages infect the bacterial pathogen, as has happened previously in a compassionate use case against *Mycobacterium abscessus* [49].

Most bacteriophages identified as infecting *P. aeruginosa* belong to the *Caudovirales* order, which has double-stranded DNA (dsDNA) and a head-and-tail shape. The three known families of bacteriophages in the *Caudovirales* are the *Myoviridae*, which has a contractile tail, the *Podoviridae*, which has a short and stubby tail, and the *Siphoviridae*, which has a long, flexible tail [50]. However, the taxonomic changes implemented by the Bacterial Viruses Subcommittee (BVS) of the International Committee on Taxonomy of Viruses (ICTV) have abolished the morphology-based families (*Myoviridae*, *Podoviridae,* and *Siphoviridae*) and removed the order *Caudovirales*, with it being replaced by the class *Caudoviricetes* to group all tailed bacterial and archaeal viruses with icosahedral capsids and dsDNA [51]. According to the ICTV’s classification, the class *Caudoviricetes* includes seven orders, 63 families, 109 subfamilies, 1360 genera, and 4079 species. The bacteriophage F1Pa belongs to the genus *Hollowayvirus*. This genus used to be included in the *Caudovirales* order and *Podoviridae* family [50]; but due to the recent taxonomy modifications, it has been classified in the *Caudoviricetes* class as an independent genus without a family [51]. The ICTV only includes two species within this genus: *Hollowayvirus F116* and *H66*. However, in the National Center for Biotechnology Information (NCBI) taxonomy browser, 35 unclassified species can be found that are probably *Hollowayvirus* [52]. The most recent *Hollowayvirus* bacteriophage described against *P. aeruginosa* is EPa33 [53]; that has been identified as a *Hollowayvirus phage* active against *Pseudomonas stutzeri* [54].

Bacteriophage tolerance thresholds to pH and temperature stability have been demonstrated by several research papers. Different ranges of pH stability are crucial to bacteriophages’ use in different clinical models such as gastrointestinal, respiratory, and urinary tract infections. F1Pa showed high stability at pHs of 4.5, 7.4, and 8; but it was not stable at pH 1. These results revealed that F1Pa phage was not suitable for oral administration due to it not being stable at the stomach’s pH (pH 1) [55], at least in its natural form. Gastrointestinal capsules could be considered as an oral form of administration [56,57,58]. Also, different ranges of thermal stability are important in determining the optimum temperature at which bacteriophages are best stored, and if phages are stable at the physiological temperature of the human body (37 °C). The optimum storage temperature for F1Pa was 4 °C. But its viability was slightly reduced at 37 °C, 21 °C, −20 °C, and −80 °C, and it was unstable at 60 °C after one week. There are no stability studies about other *Hollowayvirus*, but the thermal and pH stability of F1Pa is consistent with the stability described for *Podoviridae* phages infecting *P. aeruginosa* [40,41,43,54,59,60,61,62,63]. The complement system is a crucial component of the human innate immune system, which fights bacteria and viruses and may interact with bacteriophages, as has been previously described [64,65]. As can be seen in the results, human serum does not compromise the viability or pathogenicity of F1Pa, similarly to other *Podoviridae* bacteriophages, whose human serum stability has been investigated [66,67].

A bacteriophage can use mainly two replication strategies (lytic or lysogenic) once it has attached itself to a vulnerable host. A lytic replication cycle occurs when a phage binds to a susceptible host bacterium, inserts its genome into the cytoplasm of the host cell, and uses the host’s ribosomes to produce its protein [68]. Resources from the host cell are quickly transformed into viral genomes and capsid proteins, which come together to form several copies of the original phage. The new phage is released to infect another host cell when the original cell is either actively or passively lysed during its death [69].

The three main characteristics of bacterial phage infection are adsorption, the latency period, and the burst size. The adsorption represents the first stage of the lytic cycle of a bacteriophage, when the phage binds to a bacterium. The findings demonstrated that the adsorption rates varied significantly over time, potentially indicating a relationship with the quantity of infected bacteria or environmental variables [70]. In previous studies, at least 80% of other *Podoviridae* bacteriophages were attached to bacteria within 15 min [59,63,71], and only 57% of F1Pa phage was attached to PAO1 within 20 min, so the adsorption rate of our bacteriophage was considerably lower than previous *Podoviridae* bacteriophages. However, the adsorption rate of F1Pa was similar to other podoviruses infecting *P. aeruginosa* [60,71], although other described phages show higher adsorption rates [62,72]. The F1Pa latent period (40 min) in the one-step growth curve assay is comparable to previous studies. Many previously reported studies of different bacteriophages of the *Podoviridae* family had described a latent period of between 25 and 50 min [43,60,62,72,73] but others had described latent periods of 15 min [59], 10 min [63], and even 5 min [61]. Regarding the rise periods described in other studies for *Podoviridae* bacteriophages infecting *P. aeruginosa*, the F1Pa rise period (50 min) is shorter compared to previous studies showing 75 [61] and 100 min [60], but Baranzandeh et al. demonstrated that vB-PaeP-007 phage had an even shorter rise period (20 min) [63]. The burst size of the F1Pa phage (~400 PFUs/infected cell) was mostly larger than that previously described for *Podoviridae* phages infecting *P. aeruginosa* [43,59,60,62,63,73]. Nevertheless, PPAT (953 PFUs/infected cell), PPAY (457 PFUs/infected cell), and LP14 (785 PFU/infected cell) showed larger burst sizes than F1Pa [61,72]. A large burst size is essential for phage effectiveness [72]; phages with large bursts show advantages of selection as an antibacterial agent [74]. Interestingly, the initial dose can be increased a few hundred times quickly with bacteriophages producing huge bursts [74]. Consequently, F1Pa has a greater potential for use in wide-scale biological control of bacterial infections than other *Podoviridae* phages due to its huge burst size [43,59,60,62,63,73].

Concerning *P. aeruginosa* biofilm formation, F1Pa had activity against 76% of strong, 60% of moderate, and 45% of weak biofilm-forming testes strains. Inhibition assay results showed that clinical strains infected with F1Pa (MOI 10 and 1) had no growth up to 12 h, while normal *P. aeruginosa* clinical strains started to grow rapidly in the first 6 h. Bacterial inhibition is dose–response dependent on bacteriophage concentration, MOI. *P. aeruginosa* is a biofilm producer on abiotic surfaces (medical supplies like implants, contact lenses, and urinary catheters [75]) and biotic surfaces (epithelium of the respiratory track [2,3], wounds [6], burns [6,7,8], etc.). There are five separate phases in the development of the biofilm: initial adhesion, early attachment, young biofilm, mature biofilm, and dispersal [76]. To eradicate biofilm, bacteriophages have three distinct methods. In the first, extracellular polymeric material (EPS) is broken down by enzymes known as EPS depolymerases. These EPS depolymerases may be produced directly by bacteriophages, or bacteriophages may induce the bacterial production of them. The second mechanism is based on the internal lysis of bacteria that form biofilms after a typical phage infection. The third process, referred to as lysis from without, is independent of post-adsorption phage gene expression [77]. As can be observed, F1Pa phage can inhibit the growth of the *P. aeruginosa* reference strain PAO1 at 6 and 24 h. However, bacteria from the biofilm become resistant to the bacteriophage within 24 h. This cannot be observed in the viability of the biofilm because these bacteria disperse from the biofilm and would be in a planktonic state. These results would confirm that bacteriophage F1Pa can disintegrate *P. aeruginosa* biofilm. Regarding the *P. aeruginosa* clinical isolates, F1Pa may favor the detachment of the biofilms at 6 h due to the concentration of planktonic bacteria being higher in the presence of the bacteriophage than in the control. At 24 h, the bacteriophage also managed to stimulate the biofilm detachment or dispersal, so its viability was reduced but the number of planktonic bacteria resistant to the phage increased. Other studies had also previously described the antibiofilm activity of bacteriophages from the *Podoviridae* family against *P. aeruginosa* biofilms [59,78,79], and even their ability to disaggregate *P. aeruginosa* biofilms [80]. Corresponding to the inhibition of biofilm formation, F1Pa inhibited the biofilm formation of *P. aeruginosa* clinical isolates. These results are in line with those previously described about other *Podoviridae* phages, which were able to inhibit *P. aeruginosa* in in vitro biofilm formation [42,80,81]. Unlike this, the bacteriophage was only able to inhibit the biofilm growth and biofilm development of PAO1.

In this study, a novel bacteriophage, vB_PaeP-F1Pa, from the genus *Hollowayvirus*, class *Caudoviricetes* was discovered. It is capable of efficiently lysing clinical strains of MDR and XDR *P. aeruginosa*. F1Pa has a broad tolerance range for pH and temperature, and it can inhibit the growth and disaggregate the biofilms of *P. aeruginosa* clinical strains. In conclusion, vB_PaeP-F1Pa is a novel *Hollowayvirus* bacteriophage similar to other phages already described that, despite harboring an integrase, exhibits promising properties as an antimicrobial agent against *P. aeruginosa* strains.

## Figures and Tables

**Table 1:** *P. aeruginosa* clinical isolates from HUFJD. Antimicrobial susceptibility (S) and resistance (R) profiles for different antimicrobials: amikacin (AMK), gentamicin (GE), tobramycin (TOB), aztreonam (AZ), piperacillin/tazobactam (P/T), ceftazidime (CFT), cefepime (CEF), ceftolozane/tazobactam (C/T), imipenem (IM), meropenem (MP), ciprofloxacin (CIP), and colistin (CO).

Strain	Origin	MDR/XDR	AMK	GE	TOB	AZ	P/T	CFT	CEF	C/T	IM	MP	CIP	CO
PA1	Blood	-	S	S	S	S	S	S	S	S	S	S	S	S
PA2	Blood	-	S	S	S	S	S	S	S	S	S	S	S	S
PA3	Prosthetics	-	S	S	S	S	S	S	S	S	S	S	S	S
PA4	Blood	-	S	S	S	S	S	S	S	S	R	S	R	S
PA5	Peritoneum	-	S	S	S	R	S	S	S	S	R	R	S	S
PA6	Ulcer	-	S	S	S	S	S	S	S	S	S	S	S	S
PA7	Wound	-	S	S	S	S	S	S	S	S	S	S	S	S
PA8	Wound	-	S	S	S	S	S	S	S	S	S	S	S	S
PA9	Wound	MDR	S	R	S	R	R	R	R	R	S	R	R	S
PA10	Ulcer		S	R	S	S	S	S	S	-	S	R	S	S
PA11	Ulcer	-	S	S	S	S	S	S	S	S	S	S	S	S
PA12	Wound	-	R	S	S	S	S	S	S	R	S	S	S	S
PA13	Wound	-	S	S	S	S	R	S	R	S	S	S	R	S
PA14	Sputum	MDR	S	R	S	R	R	R	R	R	S	S	R	S
PA16	Sputum	-	S	R	-	S	R	R	R	R	R	R	S	S
PA17	Wound	-	S	S	S	R	R	S	S	S	S	S	R	S
PA18	Bronchial	XDR	S	R	-	S	R	S	S	R	R	R	R	R
PA19	Wound	-	S	S	S	S	S	S	S	S	R	S	S	R
PA20	Bronchial	MDR	S	R	S	R	R	R	R	S	R	R	R	S
PA21	Bronchial	MDR	S	R	S	R	R	R	R	R	R	-	R	S
PA22	Wound	-	S	S	S	S	S	S	S	-	R	S	S	S
PA23	Bronchial	XDR	S	R	-	S	S	S	S	R	R	R	R	R
PA24	Sputum	MDR	S	R	R	S	S	S	S	S	R	S	R	S
PA25	Sputum	MDR	S	R	R	S	R	S	S	S	R	S	R	S
PA26	Sputum	-	S	S	S	S	S	S	S	S	R	S	S	S
PA27	Bronchial	-	S	S	S	R	S	S	S	S	R	R	S	S
PA28	Sputum	XDR	S	S	-	R	R	R	R	-	R	R	R	S
PA29	Wound	-	S	S	S	-	R	S	S	S	R	R	R	S
PA30	Wound	-	S	S	S	R	S	S	S	S	R	R	R	S
PA31	Bronchial	-	S	S	S	R	R	S	S	S	R	R	S	S
PA32	Bronchial	-	S	S	S	S	S	S	S	S	R	S	S	S
PA33	Perianal	XDR	R	R	-	R	R	R	R	R	R	R	R	S
PA34	Otic	-	S	S	S	-	S	S	S	S	S	S	S	S
PA35	-	XDR	S	R	R	R	R	R	R	R	R	R	R	S
PA36	Bronchial	MDR	S	R	R	S	R	R	S	S	R	S	R	S
PA37	Urine	XDR	R	R	R	S	R	R	R	R	R	S	R	S
PA38	Urine	XDR	R	R	R	R	R	R	R	R	R	S	R	S
PA39	Urine	MDR	R	R	R	S	R	R	R	R	R	R	R	S

**Table 2:** Biofilm formation. No biofilm producer (OD ≤ 1), weak biofilm producer (1 < OD ≤ 2), moderate biofilm producer (2 < OD ≤ 4), and strong biofilm producer (4 < OD). Q1 and Q3 represent the first and third quartiles, respectively.

Strain	Biofilm Formation (Q1 to Q3)
PA1	25.79 (22.02 to 31.64)
PA2	23.95 (20.97 to 27.51)
PA3	2.01 (1.66 to 3.58)
PA4	1.59 (1.45 to 1.85)
PA5	0.85 (0.65 to 0.95)
PA6	6.88 (6.56 to 7.09)
PA7	1.24 (1.18 to 1.39)
PA8	9.54 (9.20 to 9.91)
PA9	2.32 (2.16 to 3.00)
PA10	1.65 (1.51 to 1.78)
PA11	2.40 (1.55 to 3.16)
PA12	4.31 (3.65 to 4.54)
PA13	4.61 (3.99 to 4.87)
PA14	2.55 (2.25 to 2.75)
PA16	4.91 (4.45 to 5.67)
PA17	1.55 (1.35 to 1.66)
PA18	1.89 (1.64 to 2.49)
PA19	7.39 (6.28 to 8.62)
PA20	0.83 (0.68 to 0.92)
PA21	0.84 (0.79 to 1.02)
PA22	9.73 (8.48 to 10.55)
PA23	3.85 (3.48 to 4.14)
PA24	7.50 (5.54 to 9.19)
PA25	4.59 (4.04 to 5.22)
PA26	16.69 (16.06 to 17.08)
PA27	5.82 (5.55 to 6.13)
PA28	1.21 (1.05 to 1.49)
PA29	25.40 (22.39 to 27.52)
PA30	5.42 (4.98 to 5.85)
PA31	0.64 (0.55 to 0.95)
PA32	1.04 (0.86 to 2.38)
PA33	5.59 (3.69 to 6.69)
PA34	9.96 (8.91 to 11.23)
PA35	15.26 (13.39 to 16.78)
PA36	12.30 (10.79 to 14.43)
PA37	1.68 (1.15 to 3.07)
PA38	1.24 (0.81 to 1.68)
PA39	8.03 (6.56 to 9.08)
PAO1	20.93 (15.62 to 26.86)

## Data Availability

The phage genome sequence was deposited in GenBank under accession number PP735386.

## Supplementary Materials

The following supporting information can be downloaded at: https://www.mdpi.com/article/10.3390/antibiotics13060523/s1, Figure S1: Protein structure of the vB_PaeP-F1Pa bacteriophage integrase obtained using Alphafold2. Location of conserved domains and active sites is indicated below the structure. Asterisk indicates mutated site as compared with sequence WP_033937549; Table S1. Pairwise comparison of Dunn’s test between bacteriophage F1Pa (PFU/mL) absorption and the different time points of the absorption curve; Table S2. Pairwise comparison of Dunn’s test between bacteriophage F1Pa (PFU/mL) particle release and the different time points of the burst size curve; Table S3. Pairwise comparison of Dunn’s test between the absorbance of *P. aeruginosa* clinical isolate PA24 and the different multiplicity of infection (MOIs) of bacteriophage F1Pa at 6 h; Table S4. Pairwise comparison of Dunn’s test between the absorbance of *P. aeruginosa* clinical isolate PA24 and the different multiplicity of infection (MOIs) of bacteriophage F1Pa at 12 h; Table S5. Pairwise comparison of Dunn’s test between the absorbance of *P. aeruginosa* clinical isolate PA24 and the different multiplicity of infection (MOIs) of bacteriophage F1Pa at 24 h; Table S6. Pairwise comparison of Dunn’s test between the absorbance of *P. aeruginosa* clinical isolate PA24 and the different multiplicity of infection (MOIs) of bacteriophage F1Pa at 36 h; Table S7. Pairwise comparison of Dunn’s test between the absorbance of *P. aeruginosa* clinical isolate PA35 and the different multiplicity of infection (MOIs) of bacteriophage F1Pa at 6 h; Table S8. Pairwise comparison of Dunn’s test between the absorbance of *P. aeruginosa* clinical isolate PA35 and the different multiplicity of infection (MOIs) of bacteriophage F1Pa at 12 h; Table S9. Pairwise comparison of Dunn’s test between the absorbance of *P. aeruginosa* clinical isolate PA35 and the different multiplicity of infection (MOIs) of bacteriophage F1Pa at 24 h; Table S10. Pairwise comparison of Dunn’s test between the absorbance of *P. aeruginosa* clinical isolate PA35 and the different multiplicity of infection (MOIs) of bacteriophage F1Pa at 36 h; Table S11. Pairwise comparison of Dunn’s test between the absorbance of *P. aeruginosa* clinical isolate PA36 and the different multiplicity of infection (MOIs) of bacteriophage F1Pa at 6 h; Table S12. Pairwise comparison of Dunn’s test between the absorbance of *P. aeruginosa* clinical isolate PA36 and the different multiplicity of infection (MOIs) of bacteriophage F1Pa at 12 h; Table S13. Pairwise comparison of Dunn’s test between the absorbance of *P. aeruginosa* clinical isolate PA36 and the different multiplicity of infection (MOIs) of bacteriophage F1Pa at 24 h; Table S14. Pairwise comparison of Dunn’s test between the absorbance of *P. aeruginosa* clinical isolate PA36 and the different multiplicity of infection (MOIs) of bacteriophage F1Pa at 36 h.

## References

[1] Lachiewicz A.M., Hauck C.G., Weber D.J., Cairns B.A., Van Duin D. (2017). Bacterial infections after burn injuries: Impact of multidrug resistance. Clin. Infect. Dis..

[2] Weber D.J., Rutala W.A., Sickbert-Bennett E.E., Samsa G.P., Brown V., Niederman M.S. (2007). Microbiology of ventilator-associated pneumonia compared with that of hospital-acquired pneumonia. Infect. Control Hosp. Epidemiol..

[3] Rello J., Borgatta B., Lisboa T. (2013). Risk factors for *Pseudomonas aeruginosa* pneumonia in the early twenty-first century. Intensive Care Med..

[4] Al-Hasan M.N., Wilson J.W., Lahr B.D., Eckel-Passow J.E., Baddour L.M. (2008). Incidence of *Pseudomonas aeruginosa* bacteremia: A population-based study. Am. J. Med..

[5] Zarza V.M.P., Mordani S.M., Maldonado A.M., Hernández D.Á., Georgina S.G.S., Vázquez-López R. (2019). *Pseudomonas aeruginosa*: Pathogenicity and antimicrobial resistance in urinary tract infection. Rev. Chilena Infectol..

[6] Estahbanati H.K., Kashani P.P., Ghanaatpisheh F. (2002). Frequency of *Pseudomonas aeruginosa* serotypes in burn wound infections and their resistance to antibiotics. Burns.

[7] McManus A.T., Mason A.D., McManus W.F., Pruitt B.A. (1985). Twenty-five year review of *Pseudomonas aeruginosa* bacteremia in a burn center. Eur. J. Clin. Microbiol..

[8] Gang R.K., Bang R.L., Sanyal S.C., Mokaddas E., Lari A.R. (1999). *Pseudomonas aeruginosa* septicaemia in burns. Burns.

[9] Kwiecinska-Piróg J., Przekwas J., Majkut M., Skowron K., Gospodarek-Komkowska E. (2020). Biofilm formation reducing properties of manuka honey and propolis in proteus mirabilis rods isolated from chronic wounds. Microorganisms.

[10] Saltoglu N., Ergonul O., Tulek N., Yemisen M., Kadanali A., Karagoz G., Batirel A., Ak O., Sonmezer C., Eraksoy H. (2018). Influence of multidrug resistant organisms on the outcome of diabetic foot infection. Int. J. Infect. Dis..

[11] Saltoglu N., Surme S., Ezirmik E., Kadanali A., Kurt A.F., Sahin Ozdemir M., Ak O., Altay F.A., Acar A., Cakar Z.S. (2023). The effects of antimicrobial resistance and the compatibility of initial antibiotic treatment on clinical outcomes in patients with diabetic foot infection. Int. J. Low. Extrem. Wounds.

[12] Saltoglu N., Yemisen M., Ergonul O., Kadanali A., Karagoz G., Batirel A., Ak O., Eraksoy H., Cagatay A., Vatan A. (2015). Predictors for limb loss among patient with diabetic foot infections: An observational retrospective multicentric study in Turkey. Clin. Microbiol. Infect..

[13] Pires D.P., Vilas Boas D., Sillankorva S., Azeredo J. (2015). Phage therapy: A step forward in the treatment of *Pseudomonas aeruginosa* infections. J. Virol..

[14] Hraiech S., Brégeon F., Rolain J. (2015). Bacteriophage-based therapy in cystic infections: Rationale and current status. Drug Des. Devel Ther..

[15] Costerton J.W., Stewart P.S., Greenberg E.P. (1998). Bacterial biofilms: A common cause of persistent infections. Annu. Rev. Plant Physiol. Plant Mol. Biol..

[16] Lewis K. (2001). Riddle of biofilm resistance. Antimicrob. Agents Chemother..

[17] Breidenstein E.B.M., de la Fuente-Núñez C., Hancock R.E.W. (2011). Pseudomonas Aeruginosa: All Roads Lead to Resistance. Trends Microbiol..

[18] López-Causapé C., Cabot G., del Barrio-Tofiño E., Oliver A. (2018). The versatile mutational resistome of pseudomonas aeruginosa. Front. Microbiol..

[19] Hill C., Mills S., Ross R.P. (2018). Phages & antibiotic resistance: Are the most abundant entities on earth ready for a comeback?. Future Microbiol..

[20] Potron A., Poirel L., Nordmann P. (2015). Emerging broad-spectrum resistance in *Pseudomonas aeruginosa* and *Acinetobacter baumannii*: Mechanisms and epidemiology. Int. J. Antimicrob. Agents.

[21] Eichenberger E.M., Thaden J.T. (2019). Epidemiology and mechanisms of resistance of extensively drug resistant Gram-negative bacteria. Antibiotics.

[22] Munita J.M., Arias C.A. (2016). Mechanisms of antibiotic resistance. Microbiol. Spectr..

[23] Pérez A., Gato E., Pérez-Llarena J., Fernández-Cuenca F., José Gude M., Ovia M., Eugenia Pachón M., Garnacho J., Gonzá lez V., Pascual L. (2019). High incidence of MDR and XDR *Pseudomonas aeruginosa* isolates obtained from patients with ventilator-associated pneumonia in Greece, Italy and Spain as part of the MagicBullet clinical trial. J. Antimicrob. Chemother..

[24] del Barrio-Tofiño E., Zamorano L., Cortes-Lara S., López-Causapé C., Sá nchez-Diener I., Cabot G., Bou G., Martínez-Martínez L., Oliver A. (2019). Spanish nationwide survey on *Pseudomonas aeruginosa* antimicrobial resistance mechanisms and epidemiology. J. Antimicrob. Chemother..

[25] Tomczyk S., Zanichelli V., Grayson M.L., Twyman A., Abbas M., Pires D., Allegranzi B., Harbarth S. (2019). Clinical infectious diseases control of carbapenem-resistant Enterobacteriaceae, *Acinetobacter baumannii*, and *Pseudomonas aeruginosa* in healthcare facilities: A systematic review and reanalysis of quasi-experimental studies. Clin. Infect. Dis..

[26] Fong S.A., Drilling A.J., Ooi M.L., Paramasivan S., Finnie J.W., Morales S., Psaltis A.J., Vreugde S., Wormald P.J. (2019). Safety and efficacy of a bacteriophage cocktail in an in vivo model of *Pseudomonas aeruginosa* sinusitis. Transl. Res..

[27] Holloway B.W., Egan J.B., Monk M. (1960). Lysogeny in *Pseudomonas aeruginosa*. Aust. J. Exp. Biol. Med. Sci..

[28] Kellenberger E., Séchaud J. (1957). Electron microscopical studies of phage multiplication. Virology.

[29] Drilling A., Morales S., Jardeleza C., Vreugde S., Speck P., Wormald P.J. (2014). Bacteriophage reduces biofilm of *Staphylococcus aureus* ex vivo isolates from chronic rhinosinusitis patients. Am. J. Rhinol. Allergy.

[30] Clokie M.R.J., Kropinski A.M., Lavigne R. (2018). Bacteriophages: Methods and protocols-Volume III. Methods in Molecular Biology.

[31] Kalyaanamoorthy S., Minh B.Q., Wong T.K., Von Haeseler A., Jermiin L.S. (2017). ModelFinder: Fast model selection for accurate phylogenetic estimates Europe PMC funders group. Nat. Methods.

[32] Nguyen L.-T., Schmidt H.A., Von Haeseler A., Minh B.Q. (2014). IQ-TREE: A fast and effective stochastic algorithm for estimating maximum-likelihood phylogenies. Mol. Biol. Evol..

[33] Chibeu A., Ceyssens P.J., Hertveldt K., Volckaert G., Cornelis P., Matthijs S., Lavigne R. (2009). The adsorption of pseudomonas aeruginosa bacteriophage PhiKMV is dependent on expression regulation of type IV pili genes. FEMS Microbiol. Lett..

[34] Zurabov F., Zhilenkov E. (2021). Characterization of four virulent *Klebsiella pneumoniae* bacteriophages, and evaluation of their potential use in complex phage preparation. Virol. J..

[35] Okoliegbe I.N., Hijazi K., Cooper K., Ironside C., Gould I.M. (2021). Antimicrobial synergy testing: Comparing the tobramycin and ceftazidime gradient diffusion methodology used in assessing synergy in cystic fibrosis-derived multidrug-resistant *Pseudomonas aeruginosa*. Antibiotics.

[36] Stepanović S., Vuković D., Hola V., Di Bonaventura G., Djukić S., Ćirković I., Ruzicka F. (2007). Quantification of biofilm in microtiter plates: Overview of testing conditions and practical recommendations for assessment of biofilm production by Staphylococci. APMIS.

[37] Turner D., Kropinski A.M., Adriaenssens E.M. (2021). A roadmap for genome-based phage taxonomy. Viruses.

[38] Hyman P., Abedon S.T. (2009). Practical methods for determining phage growth parameters. Methods Mol. Biol..

[39] Hyman P., Abedon S.T. (2010). Bacteriophage host range and bacterial resistance. Adv. Appl. Microbiol..

[40] Yin Y., Wang X., Mou Z., Ren H., Zhang C., Zou L., Liu H., Liu W., Liu Z. (2022). Characterization and genome analysis of *Pseudomonas aeruginosa* phage VB_PaeP_Lx18 and the antibacterial activity of its lysozyme. Arch. Virol..

[41] Zhang Y., Meng B., Wei X., Li Y., Wang X., Zheng Y., Wang C., Cui L., Zhao X. (2021). Evaluation of phage therapy for pulmonary infection of mouse by liquid aerosol-exposure *Pseudomonas aeruginosa*. Infect. Drug Resist..

[42] Knezevic P., Obreht D., Curcin S., Petrusic M., Aleksic V., Kostanjsek R., Petrovic O. (2011). Phages of *Pseudomonas aeruginosa*: Response to environmental factors and in vitro ability to inhibit bacterial growth and biofilm formation. J. Appl. Microbiol..

[43] Alvi I.A., Asif M., Tabassum R., Aslam R., Abbas Z., Rehman S. (2020). ur RLP, a bacteriophage of the family Podoviridae, rescues mice from bacteremia caused by multi-drug-resistant *Pseudomonas aeruginosa*. Arch. Virol..

[44] Quirós P., Colomer-Lluch M., Martínez-Castillo A., Miró E., Argente M., Jofre J., Navarro F., Muniesa M. (2014). Antibiotic resistance genes in the bacteriophage DNA fraction of human fecal samples. Antimicrob. Agents Chemother..

[45] McCullor K., Postoak B., Rahman M., King C., Michael McShana W. (2018). Genomic sequencing of high-efficiency transducing streptococcal bacteriophage A25: Consequences of escape from lysogeny. J. Bacteriol..

[46] Chaturongakul S., Ounjai P. (2014). Phage-host interplay: Examples from tailed phages and Gram-negative bacterial pathogens. Front. Microbiol..

[47] Mahdavi S., Sadeghi M., Shokri R., Sadegh B. (2022). The role of bacteriophages as important reservoirs of extended-spectrum beta-lactamase genes in Azerbaijan hospitals. Microb. Drug Resist..

[48] Anomaly J. (2020). The future of phage: Ethical challenges of using phage therapy to treat bacterial infections. Public. Health Ethics.

[49] Dedrick R.M., Guerrero-Bustamante C.A., Garlena R.A., Russell D.A., Ford K., Harris K., Gilmour K.C., Soothill J., Jacobs-Sera D., Schooley R.T. (2019). Engineered bacteriophages for treatment of a patient with a disseminated drug-resistant *Mycobacterium abscessus*. Nat. Med..

[50] Lefkowitz E.J., Dempsey D.M., Hendrickson R.C., Orton R.J., Siddell S.G., Smith D.B. (2018). Virus taxonomy: The database of the International Committee on Taxonomy of Viruses (ICTV). Nucleic Acids Res..

[51] Walker P.J., Siddell S.G., Lefkowitz E.J., Mushegian A.R., Adriaenssens E.M., Alfenas-Zerbini P., Dempsey D.M., Dutilh B.E., García M.L., Curtis Hendrickson R. (2022). Recent changes to virus taxonomy ratified by the International Committee on Taxonomy of Viruses (2022). Arch. Virol..

[52] Schoch C.L., Ciufo S., Domrachev M., Hotton C.L., Kannan S., Khovanskaya R., Leipe D., McVeigh R., O’Neill K., Robbertse B. (2020). NCBI taxonomy: A comprehensive update on curation, resources and tools. Database.

[53] Campbell R.A., Farlow J., Freyberger H.R., He Y., Ward A.M., Ellison D.W., Getnet D., Swierczewski B.E., Nikolich M.P., Filippov A.A. (2021). Genome sequences of 17 diverse *Pseudomonas aeruginosa* phages. Microbiol. Resour. Announc..

[54] Liu X., Feng Z., Fan X., Nie Y., Wu X.L. (2021). Isolation and characterization of the novel *Pseudomonas stutzeri* bacteriophage 8P. Arch. Virol..

[55] Dąbrowska K. (2019). Phage therapy: What factors shape phage pharmacokinetics and bioavailability? Systematic and critical review. Med. Res. Rev..

[56] Khanal D., Chang R.Y.K., Hick C., Morales S., Chan H.K. (2021). Enteric-coated bacteriophage tablets for oral administration against gastrointestinal infections. Int. J. Pharm..

[57] Richards K., Malik D.J. (2021). Bacteriophage encapsulation in Ph-responsive core-shell capsules as an animal feed additive. Viruses.

[58] Lorenzo-Rebenaque L., Malik D.J., Catalá-Gregori P., Marin C., Sevilla-Navarro S. (2021). In Vitro and in vivo gastrointestinal survival of non-encapsulated and microencapsulated salmonella bacteriophages: Implications for bacteriophage therapy in poultry. Pharmaceuticals.

[59] Kwiatek M., Parasion S., Rutyna P., Mizak L., Gryko R., Niemcewicz M., Olender A., Łobocka M. (2017). Isolation of bacteriophages and their application to control pseudomonas aeruginosa in planktonic and biofilm models. Res. Microbiol..

[60] Yu X., Xu Y., Gu Y., Zhu Y., Liu X. (2017). Characterization and genomic study of “PhiKMV-Like” phage PAXYB1 infecting *Pseudomonas aeruginosa*. Sci. Rep..

[61] Shi X., Zhao F., Sun H., Yu X., Zhang C., Liu W., Pan Q., Ren H. (2020). Characterization and complete genome analysis of *Pseudomonas aeruginosa* bacteriophage VB_PaeP_LP14 belonging to genus *Litunavirus*. Curr. Microbiol..

[62] Namonyo S., Carvalho G., Guo J., Weynberg K.D. (2022). Novel bacteriophages show activity against selected Australian clinical strains of *Pseudomonas aeruginosa*. Microorganisms.

[63] Barazandeh M., Shahin K., Hedayatkhah A., Komijani M., Mansoorianfar M. (2021). Characterization of a novel bullet-shaped lytic bacteriophage against extensively drug-resistant *Pseudomonas aeruginosa* isolated from human and domestic sources. Vet. Res. Forum.

[64] Jerne N.K. (1956). The presence in normal serum of specific antibody against bacteriophage T4 and its increase during the earliest stages of immunization. J. Immunol..

[65] Jerne N.K. (1952). Bacteriophage inactivation by antiphage serum diluted in distilled water. Nature.

[66] Egido J.E., Dekker S.O., Toner-Bartelds C., Lood C., Rooijakkers S.H.M., Bardoel B.W., Haas P.-J. (2023). Human complement inhibits myophages against *Pseudomonas aeruginosa*. Viruses.

[67] Van Nieuwenhuyse B., Van der Linden D., Chatzis O., Lood C., Wagemans J., Lavigne R., Schroven K., Paeshuyse J., de Magnée C., Sokal E. (2022). Bacteriophage-antibiotic combination therapy against extensively drug-resistant *Pseudomonas aeruginosa* infection to allow liver transplantation in a toddler. Nat. Commun..

[68] Landa K.J., Mossman L.M., Whitaker R.J., Rapti Z., Clifton S.M. (2022). Phage-antibiotic synergy inhibited by temperate and chronic virus competition. Bull. Math. Biol..

[69] Kasman L.M., Porter L.D. (2022). Bacteriophages. Brenner’s Encyclopedia of Genetics.

[70] Gibson S.B., Green S.I., Liu C.G., Salazar K.C., Clark J.R., Terwilliger A.L., Kaplan H.B., Maresso A.W., Trautner B.W., Ramig R.F. (2019). Constructing and characterizing bacteriophage libraries for phage therapy of human infections. Front. Microbiol..

[71] Putzeys L., Poppeliers J., Boon M., Lood C., Vallino M., Lavigne R. (2023). Transcriptomics-driven characterization of LUZ100, a T7-like *Pseudomonas* phage with temperate features. mSystems.

[72] Yuanyuan N., Xiaobo Y., Shang W., Yutong Y., Hongrui Z., Chenyu L., Bin X., Xi Z., Chen Z., Zhiqiang S. (2022). Isolation and characterization of two homolog phages infecting *Pseudomonas aeruginosa*. Front. Microbiol..

[73] Zhang F., Huang K., Yang X., Sun L., You J., Pan X., Cui X., Yang H. (2018). Characterization of a novel lytic Podovirus O4 of *Pseudomonas aeruginosa*. Arch. Virol..

[74] Xu Y., Yu X., Gu Y., Huang X., Liu G., Liu X. (2018). Characterization and genomic study of phage VB_EcoS-B2 infecting multidrug-resistant *Escherichia coli*. Front. Microbiol..

[75] Ghafoor A., Hay I.D., Rehm B.H.A. (2011). Role of exopolysaccharides in *Pseudomonas aeruginosa* biofilm formation and architecture. Appl. Environ. Microbiol..

[76] Tam M., Thi T., Wibowo D., Rehm B.H.A. (2020). *Pseudomonas aeruginosa* biofilms. Int. J. Mol. Sci..

[77] Chan B., Abedon S. (2015). Bacteriophages and their enzymes in biofilm control. Curr. Pharm. Des..

[78] Amgarten D., Martins L.F., Lombardi K.C., Antunes L.P., Paula A., De Souza S., Gonçalves Nicastro G., Watanabe Kitajima E., Bento Quaggio R., Upton C. (2017). Three novel *Pseudomonas* phages isolated from composting provide insights into the evolution and diversity of tailed phages. BMC Genom..

[79] Mendes J.J., Leandro C., Mottola C., Barbosa R., Silva F.A., Oliveira M., Vilela C.L., Melo-Cristino J., Górski A., Pimentel M. (2014). In vitro design of a novel lytic bacteriophage cocktail with therapeutic potential against organisms causing diabetic foot infections. J. Med. Microbiol..

[80] Ahiwale S., Tamboli N., Thorat K., Kulkarni R., Ackermann H., Kapadnis B. (2011). In vitro management of hospital *Pseudomonas aeruginosa* biofilm using indigenous T7-like lytic phage. Curr. Microbiol..

[81] Pei R., Lamas-Samanamud G.R. (2014). Inhibition of biofilm formation by T7 bacteriophages producing quorum-quenching enzymes. Appl. Environ. Microbiol..

