# Supplementary material for: A Novel Bacteriophage Infecting Multi-Drug- and Extended-Drug-Resistant Pseudomonas aeruginosa Strains
Source: Antibiotics (Basel). 2024 Jun 3;13(6):523. doi: 10.3390/antibiotics13060523 (PMC11200629; doi:10.3390/antibiotics13060523)
Supplement: Supplementary file 1 [file antibiotics-13-00523-s001.zip › antibiotics-2984677-supplementary.pdf]

Supplementary Material

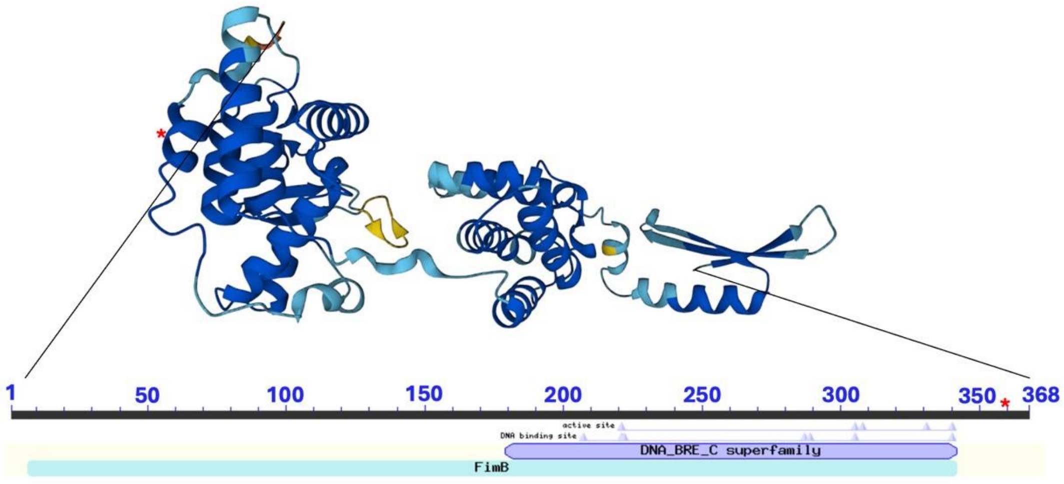

Figure S1. Protein structure of the vB_PaeP-F1Pa bacteriophage integrase obtained using Alphafold2. Location of conserved domains and active sites is indicated below the structure. Asterisk indicates mutated site as compared with sequence WP_033937549.

Table S1. Pairwise comparison of Dunn's test between bacteriophage F1Pa (PFU/ml) absorption and the different time points of the absorption curve.

	t=0	t=1	t=10	t=20	t=30	t=40
**t=1**	-0.311684<br>0.4174					
**t=10**	0.521548<br>0.3718	0.824639<br>0.3072				
**t=20**	1.736.528<br>0.0962	2.212.322<br>0.0404*	0.981714<br>0.2855			
**t=30**	1.803.317<br>0.0936	2.284.463<br>0.0391*	1.040.616<br>0.2845	0.072140<br>0.4712		

**t=40**	2.493.476<br>0.0443*	3.029.920<br>0.0128*	1.649.279<br>0.1040	0.817597<br>0.2895	0.745456<br>0.2992	
**t=5**	-0.467526<br>0.3734	-0.168328<br>0.4548	-0.962079<br>0.2714	-2.380.651<br>0.0363*	-2.452.792<br>0.0372*	-3.198.249<br>0.0145*

Table S2. Pairwise comparison of Dunn's test between bacteriophage F1Pa (PFU/ml) particle release and the different time points of the burst size curve.

	**t=0**	**t=10**	**t=100**	**t=110**	**t=120**	**t=20**
**t=10**	-0.244991<br>0.4493					
**t=100**	-3.523.210<br>0.0055*	-3.278.219<br>0.0045*				
**t=110**	-3.184.888<br>0.0051*	-2.939.897<br>0.0080*	0.338321<br>0.4344			
**t=120**	-3.604.874<br>0.0122*	-3.359.882<br>0.0061*	-0.081663<br>0.4798	-0.419985<br>0.4175		
**t=20**	-0.011666<br>0.4953	0.233325<br>0.4417	3.511.544<br>0.0043*	3.173.222<br>0.0049*	3.593.208<br>0.0064*	
**t=30**	-0.256657<br>0.4574	-0.011666<br>0.5018	3.266.552<br>0.0042*	2.928.231<br>0.0078*	3.348.216<br>0.0053*	-0.244991<br>0.4558
**t=40**	-0.653310<br>0.3338	-0.408319<br>0.4162	2.869.899<br>0.0089*	2.531.578<br>0.0221*	2.951.563<br>0.0082*	-0.641644<br>0.3332
**t=50**	-1.446.616<br>0.1408	-1.201.624<br>0.1865	2.076.594<br>0.0527	1.738.272<br>0.0916	2.158.258<br>0.0464*	-1.434.949<br>0.1372
**t=60**	-1.633.276<br>0.1079	-1.388.284<br>0.1463	1.889.934<br>0.0716	1.551.612<br>0.1208	1.971.597<br>0.0654	-1.621.610<br>0.1076

t=70	-2.169.924	-1.924.932	1.353.286	1.014.964	1.434.949	-2.158.258
	0.0509	0.0705	0.1492	0.2326	0.1405	0.0482*
t=80	-2.403.249	-2.158.258	1.119.960	0.781639	1.201.624	-2.391.583
	0.0302	0.0502	0.2009	0.2972	0.1904	0.0297*
t=90	-3.313.217	-3.068.226	0.209992	-0.128328	0.291656	-3.301.551
	0.0051*	0.0065*	0.4454	0.4669	0.4485	0.0047*

	**t=30**	**t=40**	**t=50**	**t=60**	**t=70**	**t=80**
**t=40**	-0.396652					
	0.4150					
**t=50**	-1.189.958	-0.793305				
	0.1863	0.2978				
**t=60**	-1.376.618	-0.979965	-0.186660			
	0.1461	0.2407	0.4490			
**t=70**	-1.913.266	-1.516.613	-0.723308	-0.536647		
	0.0701	0.1261	0.3103	0.3721		
**t=80**	-2.146.591	-1.749.939	-0.956633	-0.769973	-0.233325	
	0.0460*	0.0919	0.2447	0.2967	0.4480	
**t=90**	-3.056.560	-2.659.907	-1.866.601	-1.679.941	-1.143.293	-0.909968
	0.0062*	0.0160*	0.0732	0.1007	0.1973	0.2573

Table S3. Pairwise comparison of Dunn's test between the absorbance of *P. aeruginosa* clinical isolate PA24 and the different multiplicity of infection (MOIs) of bacteriophage F1Pa at 6 h.

	**Control**	**MOI 10**	**MOI 1**
**MOI 10**	0.204212		
	0.4191		
**MOI 1**	2.001.286	1.797.073	
	0.0454*	0.0542	
**MOI 0.1**	3.675.833	3.471.620	1.674.546

	0.0007*	0.0008*	0.0564

Table S4. Pairwise comparison of Dunn's test between the absorbance of *P. aeruginosa* clinical isolate PA24 and the different multiplicity of infection (MOIs) of bacteriophage F1Pa at 12 h.

	Control	MOI 10	MOI 1
**MOI 10**	2.694.438		
	0.0071*		
**MOI 1**	4.164.132	1.469.693	
	0.0001*	0.0850	
**MOI 0.1**	0.979795	-1.714.642	-3.184.336
	0.1636	0.0648	0.0022*

Table S5. Pairwise comparison of Dunn's test between the absorbance of *P. aeruginosa* clinical isolate PA24 and the different multiplicity of infection (MOIs) of bacteriophage F1Pa at 24 h.

	Control	MOI 10	MOI 1
**MOI 10**	2.571.964		
	0.0101*		
**MOI 1**	2.082.066	-0.489897	
	0.0280*	0.3121	
**MOI 0.1**	-1.224.744	-3.796.709	-3.306.811
	0.1324	0.0004*	0.0014*

Table S6. Pairwise comparison of Dunn's test between the absorbance of *P. aeruginosa* clinical isolate PA24 and the different multiplicity of infection (MOIs) of bacteriophage F1Pa at 36 h.

	Control	MOI 10	MOI 1
**MOI 10**	2.490.314		
	0.0128*		
**MOI 1**	1.551.343	-0.938971	
	0.0906	0.1739	

**MOI 0.1**	-1.265.569<br>0.1234	-3.755.884<br>0.0005*	-2.816.913<br>0.0073*

Table S7. Pairwise comparison of Dunn's test between the absorbance of *P. aeruginosa* clinical isolate PA35 and the different multiplicity of infection (MOIs) of bacteriophage F1Pa at 6 h.

	**Control**	**MOI 10**	**MOI 1**
**MOI 10**	2.941.306<br>0.0033*		
**MOI 1**	4.411.959<br>0.0000*	1.470.653<br>0.0707	
**MOI 0.1**	1.470.653<br>0.0848	-1.470.653<br>0.1060	-2.941.306<br>0.0049*

Table S8. Pairwise comparison of Dunn's test between the absorbance of *P. aeruginosa* clinical isolate PA35 and the different multiplicity of infection (MOIs) of bacteriophage F1Pa at 12 h.

	**Control**	**MOI 10**	**MOI 1**
**MOI 10**	3.327.947<br>0.0013*		
**MOI 1**	4.022.120<br>0.0002*	0.694173<br>0.2438	
**MOI 0.1**	1.470.013<br>0.0849	-1.857.933<br>0.0474*	-2.552.106<br>0.0107*

Table S9. Pairwise comparison of Dunn's test between the absorbance of *P. aeruginosa* clinical isolate PA35 and the different multiplicity of infection (MOIs) of bacteriophage F1Pa at 24 h.

	**Control**	**MOI 10**	**MOI 1**
**MOI 10**	2.816.913<br>0.0048*		
**MOI 1**	1.592.168	-1.224.744	

	0.0835	0.1103	
MOI 0.1	-1.469.693	-4.286.607	-3.061.862
	0.0850	0.0001*	0.0033*

Table S10. Pairwise comparison of Dunn's test between the absorbance of *P. aeruginosa* clinical isolate PA35 and the different multiplicity of infection (MOIs) of bacteriophage F1Pa at 36 h.

	Control	MOI 10	MOI 1
MOI 10	3.102.687		
	0.0058*		
MOI 1	2.612.789	-0.489897	
	0.0090*	0.3745	
MOI 0.1	0.163299	-2.939.387	-2.449.489
	0.4351	0.0049*	0.0107*

Table S11. Pairwise comparison of Dunn's test between the absorbance of *P. aeruginosa* clinical isolate PA36 and the different multiplicity of infection (MOIs) of bacteriophage F1Pa at 6 h.

	Control	MOI 10	MOI 1
MOI 10	3.307.530		
	0.0014*		
MOI 1	4.042.536	0.735006	
	0.0002*	0.2312	
MOI 0.1	1.470.013	-1.837.516	-2.572.523
	0.0849	0.0496*	0.0101*

Table S12. Pairwise comparison of Dunn's test between the absorbance of *P. aeruginosa* clinical isolate PA36 and the different multiplicity of infection (MOIs) of bacteriophage F1Pa at 12 h.

	Control	MOI 10	MOI 1
MOI 10	4.368.256		
	0.0000*		

**MOI 1**	2.980.212<br>0.0043*	-1.388.044<br>0.0826	
**MOI 0.1**	1.469.693<br>0.0850	-2.898.562<br>0.0037*	-1.510.518<br>0.0982

Table S13. Pairwise comparison of Dunn's test between the absorbance of *P. aeruginosa* clinical isolate PA36 and the different multiplicity of infection (MOIs) of bacteriophage F1Pa at 24 h.

	**Control**	**MOI 10**	**MOI 1**
**MOI 10**	2.204.540<br>0.0206*		
**MOI 1**	2.694.438<br>0.0071*	0.489897<br>0.3121	
**MOI 0.1**	-0.979795<br>0.1963	-3.184.336<br>0.0022*	-3.674.234<br>0.0007*

Table S14. Pairwise comparison of Dunn's test between the absorbance of *P. aeruginosa* clinical isolate PA36 and the different multiplicity of infection (MOIs) of bacteriophage F1Pa at 36 h.

	**Control**	**MOI 10**	**MOI 1**
**MOI 10**	3.409.614<br>0.0010*		
**MOI 1**	2.347.938<br>0.0142*	-1.061.676<br>0.1730	
**MOI 0.1**	-0.122501<br>0.4513	-3.532.115<br>0.0012*	-2.470.439<br>0.0135*
